# Autoimmune Pancreatitis Presenting as Multifocal Masses: A Rare Case Report

**DOI:** 10.7759/cureus.78955

**Published:** 2025-02-13

**Authors:** Aqsa Khan, Rizwan Mushtaq, Essam Rashad, Kamran Mushtaq, Neil Sharma

**Affiliations:** 1 Internal Medicine, Parkview Health, Fort Wayne, USA; 2 Internal Medicine, Ayub Medical College, Abbottabad, PAK; 3 Internal Medicine, Northeast Internal Medicine Associates, LaGrange, USA; 4 Division of Interventional Oncology and Surgical Endoscopy (IOSE), Parkview Health, Fort Wayne, USA

**Keywords:** autoimmune pancreatitis (aip), corticosteroids, diffuse lymphadenopathy, igg-4 related disease, multifocal mass

## Abstract

Autoimmune pancreatitis (AIP) is an uncommon inflammatory condition that can mimic pancreatic malignancy both clinically and radiologically, leading to diagnostic uncertainty. Two subtypes exist: Type 1 is linked to systemic IgG4-related disease, and Type 2 is confined to the pancreas. A 64-year-old woman was found to have submandibular and cervical lymphadenopathy during a routine physical examination. Further imaging revealed FDG-avid lymph nodes and pancreatic abnormalities, raising concern for malignancy. However, biopsy results confirmed Type 1 autoimmune pancreatitis (AIP) and IgG4-related sialadenitis. The patient was treated with corticosteroids, which resolved her symptoms and improved imaging findings. This case emphasizes the need to consider atypical presentations of AIP and highlights the effectiveness of steroid therapy in IgG4-related diseases.

## Introduction

This abstract was presented at ACG (American College of Gastroenterology) 2024 on October 29th, 2024, in Philadelphia. 

Multifocal masses in autoimmune pancreatitis (AIP) are rare and can mimic malignancy. AIP is an uncommon form of chronic pancreatitis characterized by inflammation and fibrosis [1-3]. It is generally considered a benign yet heterogeneous condition [1-2,4]. AIP is classified into two subtypes: Type 1 and Type 2 [1,5]. Type 1 AIP is a systemic disorder frequently associated with elevated IgG4 levels, whereas Type 2 AIP is primarily confined to the pancreas and lacks IgG4-positive cell infiltration [1-2,5]. Histologically, Type 1 AIP is characterized by lymphoplasmacytic periductal inflammation, while Type 2 AIP demonstrates ductal epithelial infiltration with neutrophils [1-2,5]. Type 1 AIP is the most prevalent form worldwide [1]. The exact incidence and prevalence of AIP remain unclear [1-2,4,6]. Type 1 AIP predominantly affects older individuals, typically between the ages of 60 and 70, and has a male predominance [1-5,7]. Although AIP often presents as diffuse pancreatic enlargement, multifocal masses are an unusual finding [3,8-9]. This atypical presentation can be diagnostically challenging, as it may be mistaken for malignancies such as pancreatic cancer or lymphoma [3]. We report a case of a 64-year-old woman presenting with multifocal pancreatic masses, ultimately diagnosed with AIP. This case highlights the importance of recognizing atypical manifestations of AIP to prevent misdiagnosis and avoid unnecessary interventions.

## Case presentation

**Figure 1:**
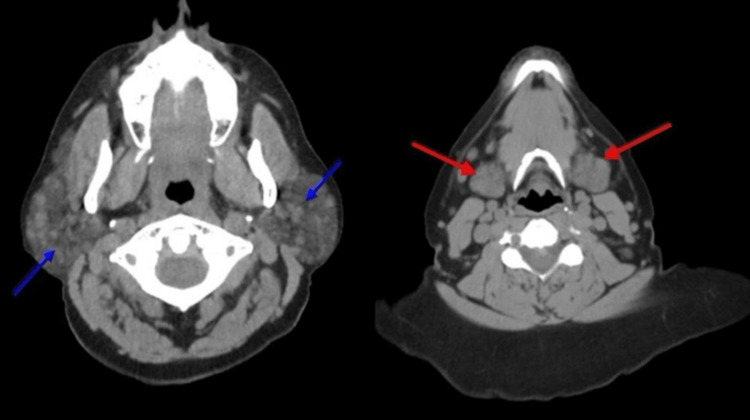
CT neck without contrast. Blue arrows showing enlarged parotid glands and innumerable lymph nodes, red arrows showing enlarged submandibular glands CT: Computed tomography

A 64-year-old female with a past medical history of asthma was found to have submandibular and anterior cervical lymphadenopathy on a routine annual physical examination. The other physical examination was unremarkable. She reported no recent illnesses. A laboratory workup, including a complete blood count (CBC), basal metabolic panel (BMP), and liver enzymes, was unremarkable. A computed tomography (CT) scan of the neck without contrast demonstrated bilateral parotid and submandibular gland enlargement with numerous lymph nodes distributed throughout both parotid and submandibular regions (Figure 1).

**Figure 2:**
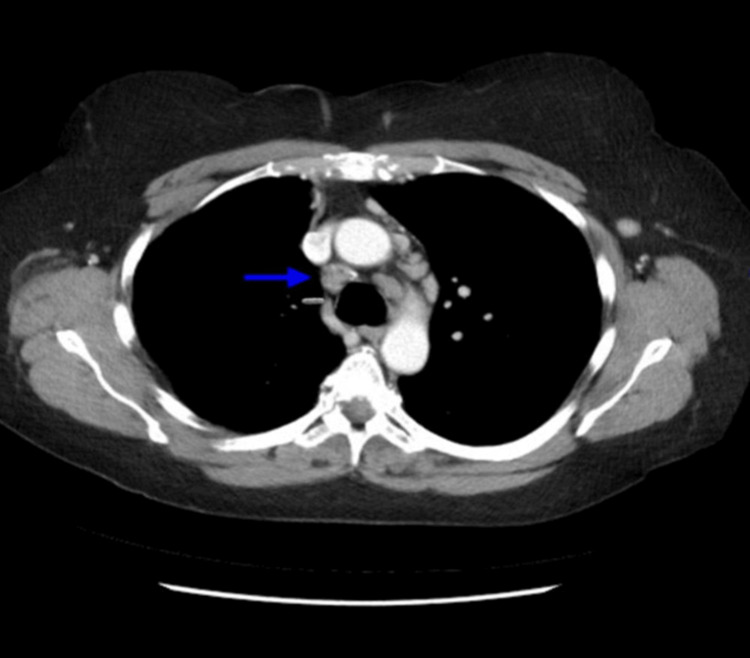
CT chest with contrast. Blue arrow showing mediastinal lymphadenopathy CT: Computed tomography

A CT scan with contrast of the chest revealed emphysematous changes and multiple mediastinal lymph nodes, some demonstrating calcifications (Figure 2).

**Figure 3:**
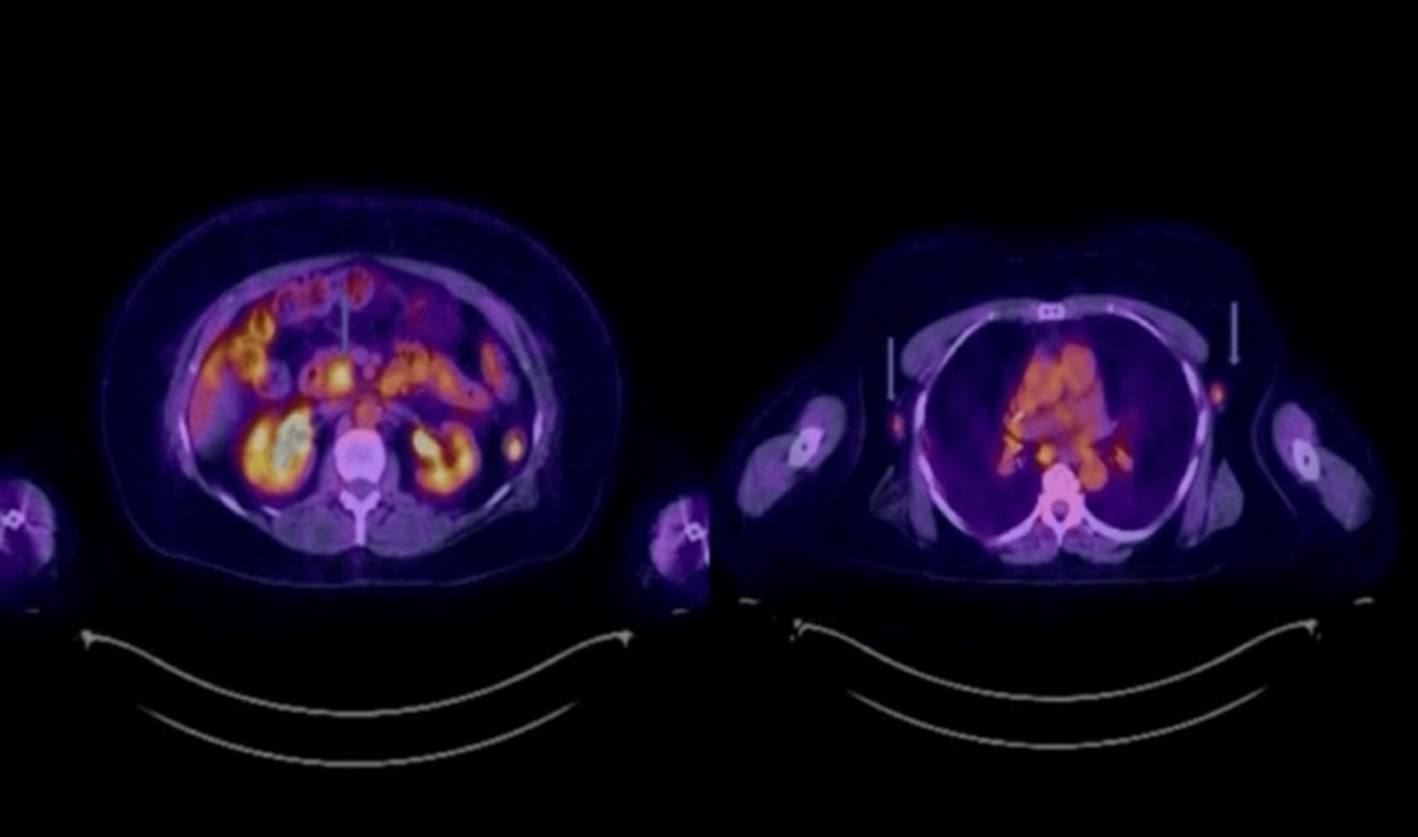
PET/CT. Blue arrows showing FDG activity in the head of the pancreas and bilateral axillary glands FDG: Focal fluorodeoxyglucose, PET: Positron emission tomography, CT: Computed tomography

Given the concern for an underlying malignant etiology of lymphadenopathy, a positron emission tomography/computed tomography (PET/CT) scan was performed, which demonstrated focal fluorodeoxyglucose (FDG) uptake within the pancreas and adjacent structures. Additionally, FDG-avid lymphadenopathy was noted in the cervical, mediastinal, and abdominal regions, raising suspicion for metastatic disease (Figure 3).

A triphasic contrast-enhanced CT scan of the abdomen and pancreas demonstrated normal pancreatic contour with no discrete mass lesions. Enhancement patterns in the arterial and venous phases were preserved. Given the abnormal FDG uptake in the pancreas on PET/CT, an endoscopic ultrasound (EUS) with fine-needle biopsy (FNB) was performed to obtain a tissue diagnosis and rule out malignancy.

EUS identified three pancreatic masses

**Figure 4:**
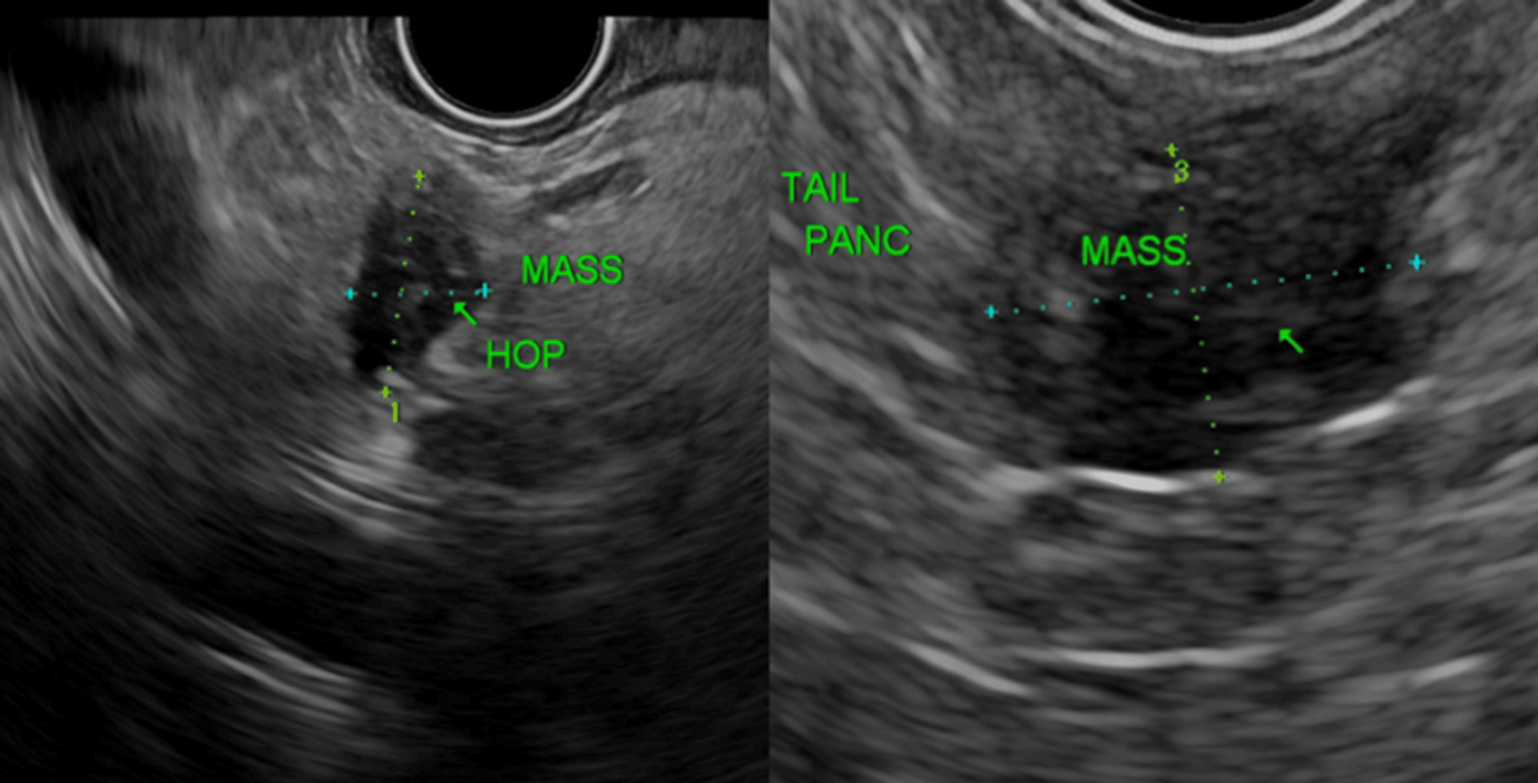
Endoscopic ultrasound (EUS) showing mass in head and tail of pancreas

Site 1: Hypoechoic, well-defined lesion with hyperechogenic focus on the tail of the pancreas. The lesion was 8.4 mm x 11 mm in size. Site 2: Mass with similar echogenicity in the superior head of the pancreas. It was 11.6 x 7.3 mm and had similar echogenicity to the lesion in the tail of the pancreas. Site 3: Third mass in the inferior head of the pancreas (poorly defined and hypoechogenic and heterogeneous in appearance). It was 19.6 mm x 11.3 mm in size. Impression was an inflammatory mass vs. carcinoma. The ampulla was normal on EUS imaging. The pancreatic duct measured 1.1 mm in the tail of the pancreas. There was sludge in the common hepatic duct. No intrahepatic ductal dilatation on EUS imaging (Figure 4).

**Figure 5:**
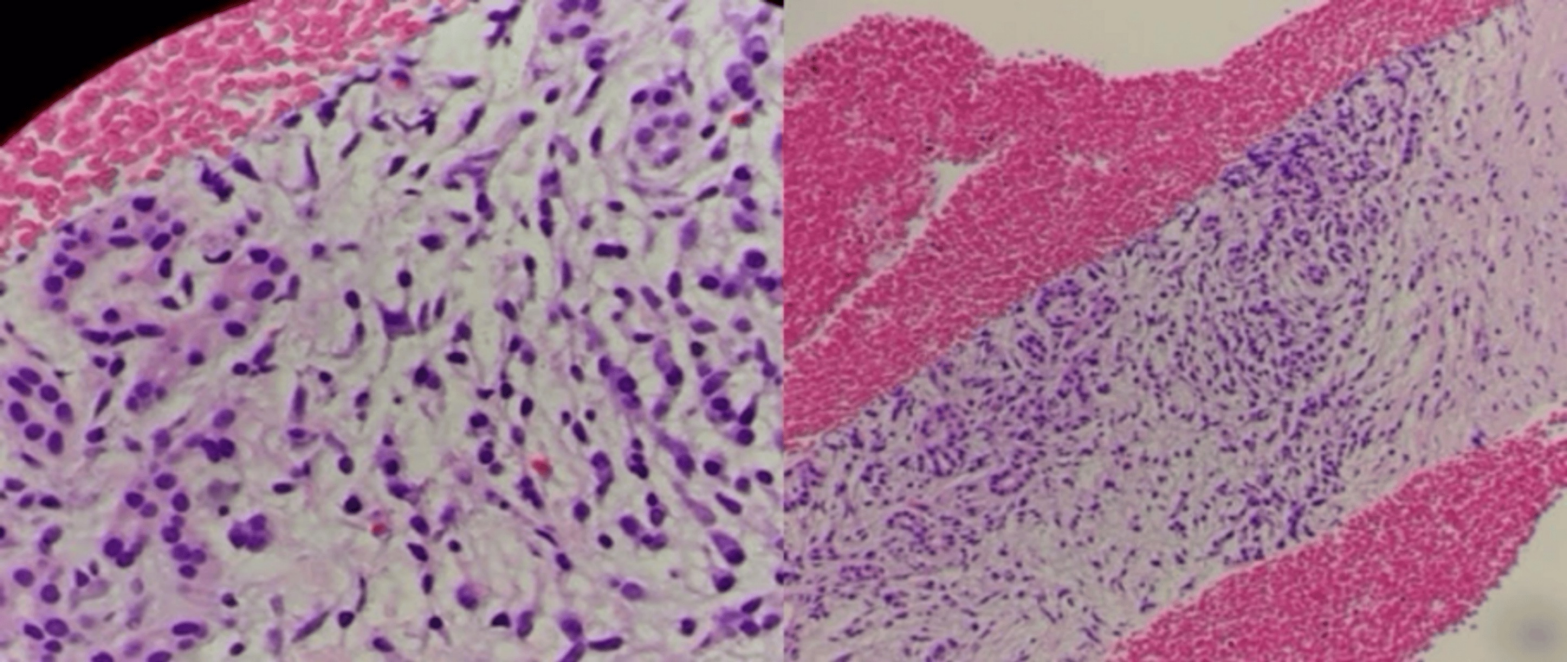
High and low power histology showing plasma cells and lymphocytes

Histopathologic evaluation of the biopsy specimens revealed features consistent with autoimmune pancreatitis, including storiform fibrosis, increased IgG4-positive plasma cells, lymphoplasmacytic inflammation, and obliterative phlebitis. No evidence of malignancy was observed (Figure 5).

To further evaluate cervical lymphadenopathy, the patient underwent an excisional biopsy of an upper left neck mass. Histopathological analysis confirmed the diagnosis of IgG4-related sialadenitis.

The patient was initiated on prednisone 40 mg daily for six weeks and then tapered by 5 mg/kg, with a favorable clinical response. On follow-up evaluation at three months, repeat CT imaging of the abdomen, pelvis, and chest demonstrated resolution of prior abnormalities without evidence of recurrent or persistent pathology.

## Discussion

The most common presenting symptom of AIP is painless jaundice/obstructive jaundice [1-2,5,10]. The obstructive jaundice is a characteristic feature of AIP [11]. Abdominal pain is most commonly associated with Type 2 AIP [1,6]. Type 1 AIP is usually associated with other immune-mediated extrapancreatic disorders like IgG4 cholangitis (autoimmune sclerosing cholangitis), mediastinal fibrosis, retroperitoneal fibrosis, tubulointerstitial disease, sclerosing sialadenitis, pulmonary nodules, and lymphadenopathy [1-2,4,6]. Patients can present with the disorders of exocrine and/or endocrine pancreas [4]. AIP (Type 2 AIP more than Type 1) can be associated with inflammatory bowel disease [1,3]. Imaging in patients with AIP can show a focal mass, which can be difficult to differentiate from pancreatic cancer.

Different diagnostic criteria/guidelines have been used for AIP [1,4,6]. One of the most commonly used diagnostic criteria in the United States is HISORt Criteria (Histology, Characteristic Imaging, Serologic Testing, Other Organ Involvement, and Response to Glucocorticoid Therapy) [1, 5-6]. The International Consensus Diagnostic Criteria (ICDC) guidelines differentiate type 1 and type 2 AIP [1]. ICDC guidelines are based on histology, imaging of pancreatic parenchyma/ducts, serology (serum IgG4 levels), other organ involvement or extrapancreatic manifestations, and response to the steroid treatment [1,6].

Histologically, type 1 AIP (lymphoplasmacytic sclerosing pancreatitis (LPSP) is characterized by periductal lymphoplasmacytic infiltration [1,5-6,10]. Other histological features include storiform fibrosis, obliterative phlebitis, and abundant (>10 cells/HPF) IgG 4 positive cells [4,6]. Type 2 is characterized by ductal granulocytic/neutrophilic infiltration along with absent (0-10 cells/HPF) IgG4-positive cells [1,5-6]. Laboratory findings include a cholestatic pattern of liver enzyme elevation [4-5,7] and hyperbilirubinemia due to strictures in the distal bile duct [7]. Total IgG and IgG4 levels are elevated in AIP [4-5]. Higher levels of IgG4 (2 times or above the upper limit of normal or >140 mg/dL) increase the specificity (> 90%) in diagnosing AIP [1,4-6].

CT and MRI usually show a diffusely enlarged pancreas with loss of lobular architecture, resulting in a sausage-shaped pancreas [1-2,4-6]. Sometimes CT and MRI can show delayed enhancement with or without rim-like enhancement [1-2,4,6]. Sometimes a focal pancreatic mass is found on pancreatic imaging, which can be difficult to differentiate from pancreatic cancer [1-2,8,12]. ERCP findings of long stricture (greater than one-third of the length of the pancreatic duct) without significant dilatation above the stricture are important characteristic features of AIP. Other findings could be multiple strictures and side branches arising from the stricture site [1-2,5]. Other occasional findings on ERCP could be stenosis of the intrapancreatic bile duct [11]. EUS and EUS-guided FNA can support the diagnosis of AIP when combined with clinical features/data [10]. EUS-FNA or EUS tract biopsy is helpful in excluding pancreatic cancer [8,9].

Both type 1 and type 2 AIP respond to steroids [1-2,4-6]. The usual treatment is prednisone 40 mg daily for four weeks followed by a 5 mg taper weekly [1,4-5]. Some patients require prolonged treatment with low-dose steroids (2.5 to 5 mg oral prednisone) [1,5]. The patient’s response to the treatment can be assessed by clinical features, radiological evaluation, and serology [1-2,4-5]. Type 1 AIP is associated with a relapse rate of 30-50%, and type 2 AIP is usually not associated with relapse [1]. For relapses, a second course of steroids is usually used [4-5]. Immunomodulators such as Rituximab can be used in patients who relapse after steroid treatment or on those who fail steroid treatment or who do not tolerate steroids [6].

## Conclusions

Autoimmune pancreatitis (AIP) can present atypically with multifocal pancreatic masses, posing a diagnostic challenge by mimicking malignancies such as pancreatic cancer or lymphoma. Early and accurate diagnosis is crucial to prevent unnecessary surgical interventions and ensure timely corticosteroid therapy, which can lead to significant clinical and radiologic improvement. This case highlights the importance of utilizing advanced imaging techniques, histopathologic analysis, and serologic markers to differentiate AIP from malignancies. Given the potential for relapse, long-term monitoring, and individualized treatment strategies are essential. The use of immunomodulators may be necessary in cases of recurrent disease or corticosteroid intolerance. Greater awareness of AIP’s variable presentations will aid clinicians in prompt recognition and management, ultimately improving patient outcomes.

## References

[1] Ketwaroo GA, Sheth S (2013). Autoimmune pancreatitis. Gastroenterol Rep (Oxf).

[2] Finkelberg DL, Sahani D, Deshpande V, Brugge WR (2006). Autoimmune pancreatitis. N Engl J Med.

[3] Hota P, Patel T, Zhao X, Jhala N, Agosto O (2018). A rare multifocal pattern of type 2 autoimmune pancreatitis with negative IgG4: A potential diagnostic pitfall that may mimic multifocal pancreatic adenocarcinoma. Case Rep Gastroenterol.

[4] Law R, Bronner M, Vogt D, Stevens T (2009). Autoimmune pancreatitis: a mimic of pancreatic cancer. Cleve Clin J Med.

[5] Nagpal SJ, Sharma A, Chari ST (2018). Autoimmune pancreatitis. Am J Gastroenterol.

[6] Grover S, Jajoo K (2016). Screening for pancreatic cancer in high-risk populations. Gastroenterol Clin North Am.

[7] Horiuchi A, Kawa S, Hamano H, Hayama M, Ota H, Kiyosawa K (2002). ERCP features in 27 patients with autoimmune pancreatitis. Gastrointest Endosc.

[8] Suzumura K, Hatano E, Uyama N (2017). Multifocal mass lesions in autoimmune pancreatitis. Case Rep Gastroenterol.

[9] Shiokawa M, Kodama Y, Hiramatsu Y (2011). Autoimmune pancreatitis exhibiting multiple mass lesions. Case Rep Gastroenterol.

[10] Farrell JJ, Garber J, Sahani D, Brugge WR (2005). EUS findings in patients with autoimmune pancreatitis. Gastroenterology.

[11] Pearson RK, Longnecker DS, Chari ST, Smyrk TC, Okazaki K, Frulloni L, Cavallini G (2003). Controversies in clinical pancreatology: autoimmune pancreatitis: does it exist?. Pancreas.

[12] Mikami K, Itoh H (2002). MR imaging of multifocal autoimmune pancreatitis in the pancreatic head and tail: a case report. Magn Reson Med Sci.

